# IVM of human immature oocytes for infertility treatment and fertility preservation

**DOI:** 10.1002/rmb2.12524

**Published:** 2023-07-11

**Authors:** Ri‐Cheng Chian, Jian‐Hua Li, Jin‐Ho Lim, Hiroaki Yoshida

**Affiliations:** ^1^ Center for Reproductive Medicine Shanghai 10th People's Hospital of Tongji University Shanghai China; ^2^ Reproductive Medical Center, Senior Department of Obstetrics and Gynecology The Seventh Medical Center of PLA General Hospital Beijing China; ^3^ Maria Fertility Hospital Seoul Korea; ^4^ Sendai ART Clinic Sendai Japan

**Keywords:** clinical application, embryos, IVF, IVM, oocytes

## Abstract

**Background:**

Thousands of healthy babies are born from in vitro maturation (IVM) procedures, but the rate of efficiency differs with the source of immature oocytes obtained. Recently, there are different IVM protocols proposed for infertility treatment and fertility preservation.

**Methods:**

Based on the literature, the clinical application for IVM of immature oocytes was summarized.

**Main findings (Results):**

Immature oocytes may be retrieved from women after priming with or without the use of follicular stimulation hormone (FSH), human chorionic gonadotrophin (hCG) or a combination of both FSH and hCG. Successful pregnancy rates with IVM technology seem to be correlated with the number of immature oocytes obtained. With the source and culture course of immature oocytes, there are various IVM protocols. IVM of immature oocytes is profoundly affected by the culture conditions, but no breakthrough has been made by improving the IVM medium itself. Thus, the clinical application of IVM technology continues to evolve.

**Conclusion:**

IVM technology is a useful technique for infertile women and fertility preservation. Mild stimulation IVF combined with IVM of immature oocytes is a viable alternative to the conventional stimulation IVF cycle treatment as it may prove to be an optimal first‐line treatment approach.

## 1 INTRODUCTION

The biological definition of oocyte maturation is the reinitiating of the first meiotic division to metaphase‐II (M‐II) stage, accompanied by cytoplasmic maturation, to successfully prepare the oocyte for fertilization and early embryonic development until the zygotic and embryonic genome activation.^1^ The LH surge triggers oocyte maturation from germinal vesicle (GV) stage to M‐II. Human immature oocytes can be matured spontaneously to M‐II stage in vitro when they were removed from the antral follicles and cultured in the proper culture media.

In vitro maturation (IVM) of human immature oocytes is a technique that was initially attempted and performed in the 1930s.^2^, ^3^ At the same time, human in vitro fertilization (IVF) started by using in vitro matured oocytes.^4^, ^5^ However, the landmark work of IVM using human immature oocytes was carried out in the 1960s,^6^, ^7^, ^8^ and the technique of in vitro fertilization (IVF) for human was established with in vitro matured oocytes.^9^, ^10^ Nevertheless, IVM of human immature oocytes was developed as a clinical procedure a few decades after the first live birth from in vitro matured oocytes,^11^ which is more than a decade after the procedure of in vivo matured oocyte following IVF.^12^

To date, assisted reproductive technologies (ARTs) have helped millions of women overcome infertility globally. Although thousands of healthy babies were born from IVM procedures, as shown in the reports by Trounson et al.^13^ and Chian et al.,^14^ their efficiencies are quite different depending on the source of immature oocytes obtained.^15^, ^16^, ^17^, ^18^ In 2021, the Practice Committees of American Society for Reproductive Medicine (ASRM) for ARTs suggested that IVM technology should no longer be considered experimental and that IVM has the potential for wide clinical application. IVM is not applicable to every patient and only those with a high antral follicle count (AFC) are considered good candidates.^19^ Recently, there have been updated information reports regarding IVM protocols on their sources of immature oocytes obtained. Therefore, the aim of this review is to share our view of the clinical applications of IVM technology.

## 2 SOURCE OF HUMAN IMMATURE OOCYTES

In mammals, including humans, transcription ceases during the final stages of oocyte growth and only resumes when the zygotic and embryonic genomes are activated after fertilization.^20^, ^21^ The oocytes can only use the stored mRNA to synthesize new proteins to support subsequent early embryonic development. Thus, the proper storage of maternal mRNA during oocyte growth is the key point for oocyte maturation through meiosis to generate mature and haploid oocytes.^22^

It is common belief that once the antrum of follicles is formed, the inside of the contained oocyte is fully grown and the size of the fully grown human oocyte is approximately 120 μm in diameter. It has been reported that the diameter of human immature oocytes ranges from 60 to 171 μm with a mean of 115 μm and an interquartile range from 107 to 124 μm, when collected from ovarian tissues for fertility cryopreservation derived from women between 14 and 41 years of age. This indicates that the diameter of human immature oocytes is a highly determining factor in the nuclear maturation of oocytes during IVM.^23^ Interestingly, it has been previously reported that human immature oocytes still grow during IVM when the immature oocytes are collected from the unstimulated ovaries with PCOS and the stimulated ovaries for ICSI cycles, indicating that different sources of human immature oocytes can have different growth profiles in vitro.^24^ Therefore, it seems that the source of immature oocytes can be considered critical for oocyte IVM, given that the proper storage of the maternal mRNA, when the immature oocytes are obtained, is important.

**FIGURE 1:**
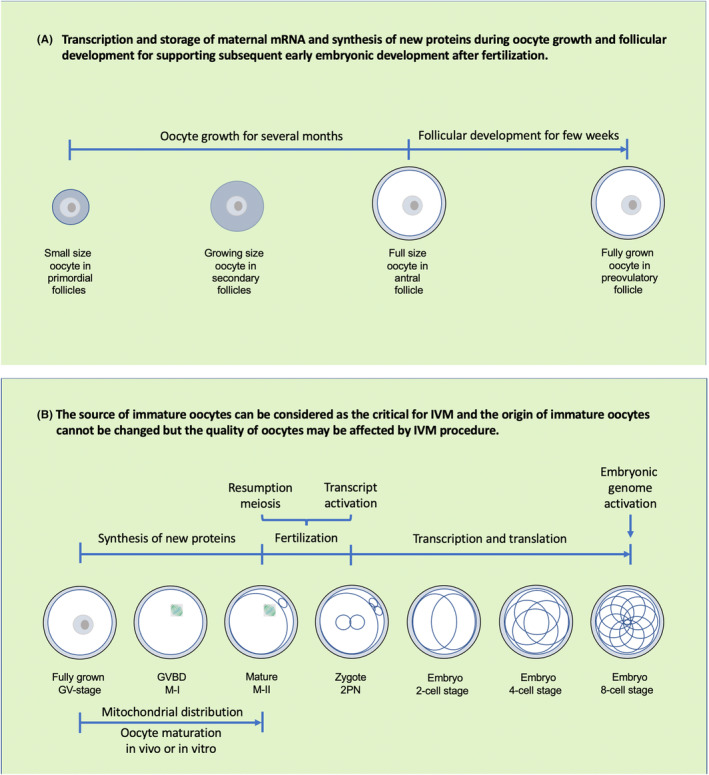
Human oocyte growth and maturation. (A) Human oocyte growth needs for several months after follicular development initiated. The transcription ceases during the final stages of oocyte growth and the oocyte can only use the stored mRNA to synthesize new proteins for supporting subsequent early embryonic development. Therefore, the proper storage of maternal mRNA during oocyte growth is the key point for oocyte maturation through meiosis to generate mature and haploid oocytes; (B) many mRNAs are stored during oocyte growth and the new proteins synthesized when the meiosis started. The maternal mRNAs and RNA‐binding proteins are mainly deposited around mitochondria in the oocytes and mitochondrial distribution may be changed during oocyte maturation. The maternal transcription is activated by fertilization, and translation may also occur during early embryonic development. However, human embryonic genome only is activated at 8‐cell stage. Therefore, the source of immature oocytes can be considered as the critical for in vitro maturation.

Recently, it also has been reported that a mitochondria‐associated membrane‐less compartment controls mitochondrial distribution and regulates maternal mRNA storage, translation, and decay to ensure fertility in mammals,^25^ indicating that maternal mRNAs and RNA‐binding proteins are mainly deposited around mitochondria in the oocytes. Although many mRNAs are stored during oocyte growth and translationally activated when the oocytes resume meiosis or after fertilization,^26^, ^27^, ^28^ it still is largely unknown that whether mitochondrial distribution is changed, and new protein synthesized during the process of oocyte meiotic maturation (Figure 1).

In addition, the clinical definition of IVM may be different than its biological definition mentioned above. It has been proposed that the clinical IVM technology should be defined as IVM of any immature oocytes, regardless of oocytes with GV stage or metaphase‐I (M‐I) stage to M‐II, because of its involvement in the IVM procedure for immature oocytes (GV and M‐I).^16^ Therefore, the definition of clinical IVM technology should be defined according to the origin of immature oocytes to help clarify the outcomes derived from the different sources of human immature oocytes. Maybe, it is interesting to mention here that commonly the mature oocytes is referred as “egg” regardless of the fact if it was matured in vivo or invitro.

IVM technology can be a useful technique for infertile women and fertility preservation. IVM is particularly effective for women with polycystic ovaries (PCO) or polycystic ovary syndrome (PCOS) – related infertility, since there are more antral follicles within the ovaries of this group of women. This group of women is sensitive to ovarian stimulation with the use gonadotropins and has an increased risk of ovarian hyperstimulation syndrome (OHSS) compared to women who have normal ovaries. Immature oocytes may be retrieved from women after priming with or without the use of FSH, hCG or a combination of both FSH and hCG. Successful pregnancy rates with IVM technique also seem to be correlated with the number of immature oocytes obtained.^29^

### 2.1 Immature oocytes from non‐primed ovaries

As mentioned above, the initial attempts of IVM for patient own immature oocytes were from women with PCO and PCOS.^13^ The immature oocytes were retrieved from the ovaries of follicular and luteal phases, and the immature oocytes were aspirated from the size of follicles at 2–10 mm in diameter. Apparently, those immature oocytes were all at GV stage and the IVM rate of those obtained immature oocytes was relatively low, reporting that the efficiency of IVM technology for infertility treatment was low. Early studies reported that the size‐dependent ability for meiotic competence depends not only on the size of the follicles but also the stage of the menstrual cycle.^30^ It means that although the immature oocytes can be retrieved from the follicles of luteal phase in the ovaries, the quality of oocytes and maturation rates may be different which compared to the immature oocytes were obtained from the follicles of follicular phase. Therefore, the size and the phase of of follicles may be important factors for the quality of immature oocytes in term of in vitro maturation potential because the maturation rates are directly related to the size and the phase of of follicles.^31^, ^32^, ^33^, ^34^, ^35^

It may be true that the clinical pregnancy and live‐birth rates were inferior with IVM technology due to the low maturity rate of immature oocytes in vitro when the immature oocytes were retrieved from non‐stimulated ovaries, especially from women with PCOS compared to the standard ovarian stimulation IVF cycles.^36^ However, it is not a suitable comparison using the different sources of mature and immature oocytes even though the oocytes were retrieved from the same infertile women with PCOS. As mentioned above, the source of mature and immature oocytes is the key for the successful treatment. Apart from the size and phase of follicles and stimulated or non‐stimulated cycles, the quality of immature oocytes is also directly related to the age of the woman.^18^

The process of pregnancy to live birth is complex, and multiple factors can affect pregnancy success in infertile women. When using IVM technology to treat infertile women, the age must be considered as a key factor affecting the outcome. The quality of in vivo matured oocytes reduces after a certain age (>35 years) and is an important factor influencing a successful ART treatment. The same theory applies to the outcome of IVM treatment, and a key obstacle to successful infertility treatment using IVM technology is the process of obtaining high quality of immature oocytes.^18^ IVM is just a technology and if used properly with high quality immature oocytes, the clinical outcomes can be the same as in vivo matured from ovarian stimulated cycles.^17^

### 2.2 Immature oocytes from primed cycles

In vivo meiotic oocyte resumption is initiated by the pre‐ovulatory LH surge. The LH surge triggers oocyte maturation from GV to M‐II stage. For infertility treatment using IVF technology, women are usually given hCG to induce the completion of follicular oocyte meiosis in natural or stimulation cycles. The final stage of oocyte meiosis can also be induced by the administration of luteinizing hormone‐releasing hormone (LHRH) agonist after follicular stimulation for IVF treatment.^37^ Therefore, without the LH surge, most of the oocytes retrieved would be at an immature GV stage, from the large size of preovulatory follicles.

The IVM technology also involves priming with FSH and/or hCG before immature oocyte retrieval. As mentioned previously, successful pregnancy rate with IVM technology correlates with the number of immature oocytes retrieved.^29^ FSH priming promotes efficient recovery of immature oocytes and maturation rate.^38^ Although a low‐dose FSH priming from the luteal phase improves the efficiency of immature oocyte recovery, the rates of maturation and fertilization are not different between women with regular menstrual cycles and women with irregular cycles of PCOS.^39^ Similarly, it has been reported that priming with r‐FSH during follicular phase before harvesting of immature oocytes from women with PCOS improves the maturational potential of oocytes and the implantation rate of the cleaved embryos.^40^ It seems that the use of FSH priming at the beginning of follicular or luteal phases enhances more follicular development and promotes the maturational competence of retrieved immature oocytes.^41^, ^42^

It has been demonstrated that the time course of oocyte maturation in vitro is hastened, and the rate of oocyte maturation is increased by priming with hCG 36 h before retrieval of immature oocytes from women with PCOS.^43^ It appears that the oocytes retrieved from follicles in response to hCG may promote the initiation of oocyte maturation in vivo, since there are LH/hCG receptors in the granulosa and cumulus cells from the antral follicles as small as 3.0 mm in diameter, suggesting a benefit to most follicles when using hCG priming before immature oocyte retrieval.^44^ Indeed, although the final rates of oocyte maturation were not different between the immature oocytes priming with and without hCG, the time course of oocyte maturation was different.^1^ Therefore, it is possible that the quality of oocytes and subsequent pregnancy rate may potentially improve by priming with hCG before immature oocyte retrieval.^45^ Interestingly, within the women with PCOS, the time course and maturation rates were different when the GV‐stage oocytes were divided into different groups based on the pattern of cumulus cells after hCG priming,^46^ indicating that hCG priming, does not only promote some oocytes initiated maturation process in vivo, but also enhances some GV‐stage oocytes from the small follicles to acquire maturational and developmental competence.^17^

Nevertheless, it has been reported a significant improvement in nuclear maturation rate of immature oocytes was observed in hCG‐primed IVM cycles with PCOS patients, but the use of hCG prior to immature oocyte retrieval did not improve the subsequent embryonic developmental competence.^47^ A review report indicated that there was no conclusive evidence that hCG‐priming influenced live birth, pregnancy, or miscarriage rates in IVM treatment.^48^ Recently, a large scale retrospective study indicated that, with 921 women using hCG‐primed IVM treatment, the live birth rate after the first embryo transfer was 31.7%, with a cumulative live birth rate from the cycles of 33.7%, suggesting that using the hCG‐priming IVM treatment for women with PCOS was feasible and resulted in acceptable live birth rates.^49^ Although a retrospective cohort study with data analysis of 324 women with PCOS reported that the trend of oocyte maturation rate was higher in hCG priming group than the non‐priming group (52.7% vs. 48.6%), no statistical significance was found.^50^ They concluded that there were no significant differences in clinical pregnancy between the groups, and the miscarriage and live birth rates were similar between the groups, indicating that hCG priming before immature oocyte retrieval may not improve clinical outcomes of IVM treatment in women with PCOS. However, it is important to point out that the clinical outcomes are affected by many factors, but the confirmed key factor is the hCG priming which improves oocyte maturation in terms of percentage and time course.^1^

Interestingly, FSH priming for a few days in combination with hCG priming 36 h before immature oocyte retrieval seems to have no additional benefit on women with PCOS for IVM treatment.^51^ It is hardly seen leading or dominant follicles in the ovaries during IVM treatment cycles from women with PCOS even though priming with FSH for a few days. However, it is practically possible to retrieve M‐I stage oocytes and sometimes M‐II stage oocytes, from the follicles 36 h after hCG priming. Therefore, it is important to mention that the situation and results will be quite different with normal menstrual cycling and PCOS ovaries after hCG priming.

With the advantages of IVM treatment, it has been applied to some infertile women with normal menstrual cycling ovaries who prefer IVM treatment. It seems that FSH priming before immature oocyte retrieval may obtain more immature oocytes in this group of patients. However, priming with fixed dose of 150 IU/day FSH for 3 days from Day 3 of the menstrual cycle without hCG priming for IVM treatment did not increase the number of oocytes obtained per aspiration and did not improve oocyte maturation and embryonic development.^52^, ^53^ A study was designed to determine whether the efficiency of IVM treatment in women with normal menstrual cycling ovaries can be improved by gonadotropin administration and the results indicated that a more favorable outcome can be obtained with a combination of FSH plus hCG priming and that FSH priming alone or hCG priming alone had no significant effect on the clinical outcomes.^54^ Recently, it has been reported that in IVM cycles, women received 3 days of FSH 100 IU/day followed by hCG 10 000 IU, and the results of live birth rate after the first transfer was 36.5%, indicating that IVM is an effective alternative compared to regular stimulating IVF cycles in women with high antral follicle count (AFC) undergoing ART treatment, while it also eliminates the risk of OHSS.^55^

### 2.3 Immature oocytes from ovarian tissue or during obstetric gynecological surgeries

Immature oocytes may be obtained from surgical materials of ovaries at follicular and luteal phases, in which human IVM technology was developed. From those immature oocytes, the first IVM babies were born.^11^ It seems that the immature oocytes derived from different phases of menstrual cycle do not affect adversely oocyte maturation in vitro and subsequent fertilization and embryonic development. The immature oocytes from obstetric and gynecological surgical materials may provide the possibility for younger women for fertility preservation with immature oocyte collection.

Recent advances in vitrification techniques have markedly improved the efficacy of oocyte cryopreservation in terms of oocyte survival and pregnancy rates as well as live birth rates that are now comparable to those achieved with fresh oocytes for IVF treatment. The number of live births from the vitrified oocytes has increased rapidly over the past two decades. To date, most live births were from in vivo matured oocytes produced from standard ovarian stimulation cycles, and only a few live births were from cryopreserved IVM oocytes.^56^, ^57^, ^58^ However, only a few pregnancies have been reported from cycles for fertility preservation of cancer patients from IVM oocytes after cryopreservation.^59^, ^60^, ^61^ Therefore, the efficiency must be considered for cryopreservation of the oocytes or embryos produced from immature oocytes after IVM.

In addition, a major question is whether immature oocytes should be cryopreserved before or after IVM. With the special structure of immature oocytes with the GV‐stage oocytes compared to mature MII stage oocytes, it has been proposed that cryopreservation at the immature GV stage may reduce damage to the oocytes from the freezing procedure.^62^ Theoretically, the use of immature GV‐stage oocytes circumvents the risk of polyploidy and aneuploidy because the chromatins are diffuse in the diplotene state of prophase‐I and are surrounded by a nuclear membrane, which may avoid spindle depolymerization.^62^ However, proper IVM of GV‐stage oocytes after freezing–thawing still is questionable. Although the survival rates seem to be improved, the quality of IVM oocytes is still major problem associated with immature oocyte freezing.^63^

With the development of vitrification techniques, it was found that there is no difference in the survival rate between oocytes vitrified at the immature GV stage and those vitrified at the mature M‐II stage.^64^ But the potential of oocyte maturation was reduced significantly by the vitrification of immature oocytes at the GV stage, suggesting that oocytes should be vitrified at the mature M‐II stage after IVM rather than at the immature GV‐stage.^65^, ^66^, ^67^ As known from the early days, the proper oocyte maturation requires cumulus cells connected with gap‐junction to oocyte cytoplasm.^8^ If the oocytes were cryopreserved at immature GV‐stage, the structure of cumulus‐oocyte complex will be destroyed during freezing and thawing procedures by the high osmolarity of freezing (or vitrification) and thawing (warming) solutions. Therefore, the process of IVM with immature oocytes will be lost the gap‐junction connection of cumulus cells with oocyte cytoplasm after freezing and thawing. In such case, the oocyte maturation rate and proper cytoplasm maturation may be affected by disconnection of the cumulus cells with the oocytes.

Furthermore, immature oocytes can be collected after Caesarean section during delivery.^1^, ^68^, ^69^ The collected immature oocytes have a relatively high maturation rate following IVM.^70^ A large group of 95 pregnant women who underwent Caesarean section were performed immature oocyte collection, and the results indicated that the high proportion of oocytes retrieved at the time of Caesarean section exhibited the capacity to undergo maturation in vitro and they can be fertilized and developed into good‐quality blastocyst stage.^71^ Interestingly, they also compared the rates of maturation, fertilization, and embryo development of in vitro‐matured human oocytes derived from Caesarean section and gynecological operation women and the results had no significant difference in the rates of maturation, fertilization, and formation of good‐quality blastocysts in oocytes obtained from these two groups, indicating that the developmental competence of immature oocytes did not differ between pregnant and non‐pregnant women.^72^ Therefore, immature oocyte collection during Caesarean section followed by IVM may provide a good option for fertility preservation for women delivered by Caesarean section. At the same time, those immature oocytes followed by IVM and vitrification might be a potential source of oocyte bank for donation to infertile women who need it.

Moreover, apart from Caesarean section, immature oocytes can also be retrieved during gynecological surgery.^72^ IVM‐surgery has proposed transvaginal retrieval of immature oocytes during endoscopic gynecological procedures.^73^, ^74^ Recently, it has been reported that a total of 93 women with refractory PCOS who underwent unstimulated IVM‐surgery were included and the clinical outcomes were analyzed for the influencing factors, indicating that unstimulated IVM‐surgery during laparoscopy and hysteroscopy procedures provided the opportunity for both spontaneous pregnancy and ART treatment.^75^

Ovarian tissue cryopreservation is the primary method of fertility preservation for prepubertal girls who are at risk of premature ovarian failure (POF). Ovarian tissue cryopreservation followed by a transplant later may be offered as a method of fertility preservation for pubertal cancer patients who cannot receive ovarian stimulation due to time constraints or contraindications. Cryopreservation of ovarian tissues would only preserve primordial and primary follicles.

It has been reported that immature oocyte retrieval from the visible antral follicles after ovarian wedge resection or oophorectomy and subsequent IVM and vitrification of those oocytes represents an additional strategy for fertility preservation, indicating that this method of fertility preservation can be offered as an adjunct to ovarian tissue cryobanking.^76^ In addition, the combination of ovarian tissue cryopreservation with immature oocytes collection from the tissue followed by IVM‐vitrification of oocytes represents a promising approach of fertility preservation for young women with mosaic turner syndrome.^77^, ^78^

## 3 IVM MEDIA FOR HUMAN IMMATURE OOCYTES

Human oocytes acquire a series of competencies during follicular development (oocyte growth and maturation) that play critical roles in fertilization and subsequent early embryonic development. Although high rates of IVM of immature oocytes may be obtained, the developmental competence of IVM oocyte is still suboptimal, as indicated by the relatively minimal development up to blastocyst stage and the poor implantation rates after transfer. Oocyte IVM is profoundly affected by culture conditions. So far, numerous data have been accumulated from studies, but the current rationale for choosing a specific medium for IVM of human immature oocytes appears to stem largely from the adaptation of the methods developed for culturing other cell types. All existing media for oocyte IVM are the base of complex culture media supplemented with different substances.

The complex culture media, including energy and nitrogen sources, vitamins, antioxidants, buffered with bicarbonate and supplemented with various sera (proteins), gonadotropins (FSH and LH), insulin, growth factors, and steroids, have been widely used in research and clinical application of immature oocyte IVM.^7^, ^8^, ^13^, ^79^, ^80^ However, it is better to supplement gonadotropins, growth factors, steroid as well as insulin hormones into IVM medium just before use, because the active half‐life of hormones and growth factors is short in the IVM media. Endogenous and exogeneous signals, such as gonadotropins, growth factors, insulin and cortisol act as upstream regulators to regulate follicular lipid metabolic homeostasis, thereby maintaining oocyte meiosis and folliculogenesis.

There are many excellent review papers that describe the base components of IVM media and the functional roles of each supplement in the IVM media. Therefore, in this review, we will not deal with the common supplements, and will briefly discuss the follicular‐fluid meiosis activating sterol (FF‐MAS) and some other factors.

FF‐MAS was found in high concentrations in follicular fluid in mammalian follicular fluid,^81^ indicating that it is involved in the control of meiosis.^82^, ^83^ Animal studies indicated that addition of FF‐MAS to the culture medium induces resumption of meiosis and it is dose‐dependent.^84^ It has also been reported that addition of FF‐MAS to IVM medium increases the synchrony of oocyte nuclear and cytoplasmic maturation.^85^, ^86^, ^87^ However, the supplement of FF‐MAS into IVM medium did not improve the results of human immature oocytes in terms of in vitro maturation and embryonic development. At the same time, the results showed high rates of embryo chromosomal abnormalities.^88^ Interestingly, the authors indicated that a majority of hCG exposed immature oocytes, which have not mature at the time of oocyte pick‐up, were defect, suggesting that FSH/hCG exposed oocytes retrieved in the GV or M‐I stage should not be used in infertility treatment.

It has been reported that FF‐MAS promotes IVM of porcine oocytes, indicating that relative expression of meiosis‐related genes was upregulated by FF‐MAS.^89^ Although the results imply that the endogenous accumulation of FF‐MAS is beneficial to resumption of meiosis in porcine oocytes and that MAPK signaling is involved in FF‐MAS‐induced meiotic resumption, the beneficial results to human immature oocytes during IVM is still questionable and no further results are available. MAS are substrates of SC4MOL and NSDHL in the cholesterol pathway and are important for normal organismal development.^90^ Central in the sterol synthesis pathway producing metabolites is essential to convert squalene to cholesterol, SC4MOL and NSDHL catalyze two sequential steps of oxidative decarboxylation of C4 methyl groups from MAS.^91^, ^92^ Therefore, an attention has been made that lipid metabolism is an important biological event in the follicular microenvironment during folliculogenesis and oocyte meiosis.^93^ Nevertheless, the conclusive results of FF‐MAS in IVM medium need to be further confirmed.

In the studies, attempts to optimize the IVM medium by supplementing Melatonin have produced relatively desirable results, including animal and human immature oocytes,^94^, ^95^, ^96^ indicating that Melatonin can promote the development of human immature oocytes retrieved from the stimulated cycles into healthy offspring by protecting mitochondrial function.^97^, ^98^ Although studies reported that the quality of IVM oocytes is affected by the different culture media and supplements, it is still unclear how some factors in the culture medium enhance oocyte maturation and quality in vitro. Such numerous studies have shown that Lysophosphatidic acid also promotes oocyte maturation in vitro, as well fertilization and embryonic development.^70^, ^99^, ^100^

Oocyte maturation in livestock animals has indicated a pre‐maturation culture strategy aiming to sustain synchronization of oocyte nuclear and cytoplasmic maturation.^101^ The idea was the use of the molecular compounds for in vitro modulation of cAMP levels within the oocytes, in which high cAMP levels are required to maintain oocytes under meiotic arrest for sufficient length of time in appropriate media, to acquire developmental capacities. Therefore, a longer culture period (pre‐maturation or “capacitation” – CAPA – culture) in the presence of C‐type natriuretic peptide (CNP), followed by IVM (CAPA‐IVM) was developed to induce the stage‐dependent maturation features.^102^ It has been reported that the CAPA‐IVM can bring significant improvements in oocyte IVM from the small antral follicles (<6 mm), which can be easily retrieved from women with a minimal ovarian stimulation.^42^ However, it seems no breakthrough has yet been made for improving the IVM medium itself. The optimal IVM media for immature oocyte should be similar to the physiological condition in follicular fluid which contains fully mature oocytes.

## 4 IVM PROTOCOLS

In order to obtain acceptable clinical outcome, the improvements have been made in IVM protocols by better preparing of patients and by optimizing the oocyte culture system.

Various IVM protocols have been proposed.^103^ As discussed above, the source of immature oocytes and culture system are the most important features for IVM technology. Based on the source of immature oocytes obtained and culture course, it can be divided into: (a) IVM with or without primed by FSH and/or hCG protocols; (b) IVM with or without prolonger culture protocols.

### 4.1 IVM without FSH and hCG priming protocol

This is classic IVM protocol for immature oocytes. In this treatment, women are not given any gonadotropins for ovarian stimulation or triggering oocyte maturation and ovulation. This protocol started with gynecological surgery materials and with women with PCOS, regardless of follicular or luteal phases.^13^ The immature oocytes are retrieved from different sizes of follicles at GV‐stage and immature oocytes were required at least 30 h of culture in vitro in order to achieve mature M‐II stage.^1^, ^7^

### 4.2 IVM with FSH priming protocol

The idea of this protocol is that using small amounts of FSH stimulates the follicles to increase the size of follicles in order to retrieve more numbers of immature oocyte and their in vitro maturational potential and developmental competence. However, there is no consensus on either the dose or the duration of FSH priming. Several trials have evaluated the utility of FSH priming with the common dosage of 150 IU FSH daily for 3–6 days, initiating from Day 2 or 3 of the menstrual cycle after a progestin withdrawal bleed followed by oocyte retrieval on cycle Day 7–10 without hCG administration.^104^ The data on the priming effects of FSH on the IVM treatment have been conflicting when compared to the IVM rate of immature oocytes obtained from without FSH primed antral follicles.

### 4.3 IVM with hCG priming protocol

The first successful application of hCG triggering in IVM was reported in 1999,^43^ where a single 10 000 IU injection was used 36 h before oocyte retrieval from women with PCOS. It has been found that the time course of oocyte maturation was hastened by hCG priming before immature oocyte retrieval from women with PCOS.^45^ In fact, the size of follicles hardly reached 10 mm in diameter after withdrawal bleed with progesterone followed by oocyte retrieval on the cycle Day 10–14 with hCG administration from patients with PCOS. More than a half of immature oocytes retrieved reached M‐II stage followed 24 h of culture in vitro. This means that some of oocytes already started the process of oocyte maturation in vivo by hCG injection within 36 h in this group of women with PCOS. Therefore, it is possible to obtain more numbers of mature oocytes, and also produce more embryos by hCG priming before immature oocyte retrieval. Although there are some arguments with hCG priming before immature oocyte retrieval from women with PCOS. Although there are some arguments with hCG priming before immature oocyte retrieval from women with PCOS, there is no doubt that the time course of oocyte IVM has been fastened and that the oocyte maturation rate was increased by hCG priming to result in better clinical outcomes.

### 4.4 IVM with FSH and hCG priming protocol

Women with PCOS can be primed with a combination of FSH and hCG for IVM treatment. A recent large retrospective study of IVM treatment of 921 women with PCOS used single hCG injection combined with FSH priming and the oocyte maturation rate was 71%. The cumulative live birth rate after one IVM cycle over a period of 12 months was 33.4%.^49^ Another study compared IVM with the use of FSH plus hCG priming versus conventional IVF in 919 women with high AFC and found that the cumulative live birth rate after one cycle of conventional IVM and IVF were comparable (39.3% vs. 49.8%) and did not find significant differences in gestational age at delivery, preterm birth rate and mean birth weight between groups.^55^ Nevertheless, it is important to mention that the timing (when) for hCG injection is the key issue for women with PCOS or high AFC, because the size of follicles is critical factor for the oocytes obtained after hCG administration. Hopefully, all in vivo matured oocytes at oocyte retrieval 36 h post hCG injection can be accounted for, as mature M‐II oocytes can be obtained from relatively small follicles (12–14 mm in diameter).

In addition, it has to be emphasized especially that the immature oocytes retrieved from relatively larger follicles in the conventional ovarian stimulation protocols may not be suitable for IVM, because those oocytes should become mature at time of oocyte retrieval 36 h after hCG injection, but they were not. This means that something was already wrong with the retrieved immature oocytes.^88^

### 4.5 IVM without prolonger culture protocol

Regardless of the source (primed with or without FSH and/or hCG) of immature oocytes obtained, the obtained immature oocytes (including GV and M‐I stage of oocytes) were cultured in vitro for 24–48 h after oocyte retrieval even though some M‐I stage became mature 5–6 h after culture in vitro. Since the size of follicles were different at the time of oocyte retrieval, the potential of maturational and early embryonic developmental might be different,^35^, ^104^ in which has been evidenced by different time course of oocyte IVM.^1^, ^15^, ^45^ The GV‐stage oocytes require at least 30 h of culture in vitro after retrieval from the follicles.^1^, ^7^, ^45^ Recently, it has been reported that IVM procedure does not affect the quality of embryos and it does not increase the aneuploidy rate of embryos compared to in vivo matured oocytes, indicating that the aneuploidy rate of embryos correlates to the age of the women.^105^

### 4.6 IVM with prolonger culture protocol

With the idea that IVM oocytes may not be synchronized on nuclear and cytoplasmic maturation,^101^ a new protocol with prolonger culture has been proposed for IVM with immature oocytes: a prominent pre‐maturation culture system, also called biphasic IVM or capacitation‐IVM (CAPA‐IVM).^42^ The biphasic IVM system consists of two steps: a pre‐IVM culturing that inhibits oocyte spontaneous meiotic resumption that maintains oocyte‐cumulus cell gap junction communication, and promotes the acquisition of oocyte developmental competence; and an IVM culturing period that induces oocyte meiotic resumption and maturation. The supplementation of cyclic adenosine monophosphate (cAMP) modulators, cyclic guanosine monophosphate modulators, 3‐isobutyl‐1‐methyl‐xanthine (IBMX), or c‐type natriuretic peptide (CNP),^42^, ^102^ was used to block meiotic resumption and maintain oocytes at the GV stage for up to 24 h until they were moved into IVM medium for 30 h.

Compared to the protocol without prolonger culture, it showed an improved IVM rate and clinical pregnancy rate, and a reduced oocytes degeneration rate among women with PCOS.^106^ In women with high AFC, the clinical pregnancy rate (50.5%) and live birth rate (35.2%) in the IVM group were comparable to those in the conventional IVF group (56.4% and 43.2%, respectively) after the first embryo transfer.^107^ Meanwhile, the use of with prolonger culture protocol did not have any significant impact on childhood physical and mental development compared with children born as a result of natural conception.^108^, ^109^ It has also been reported that no significant differences were detected in imprinted DNA methylation and gene expression from the blastocysts between conventional stimulation protocol and with prolonger IVM culture protocol.^110^ However, it must be considered that the time course with prolonger culture IVM protocol is in total 54 h of culture in vitro (24 h for inhibition and 30 h for IVM), which is significantly longer than that of without prolonger culture protocol.

## 5 EXPANDING OF IVM TECHNOLOGY

Immature oocyte retrieval followed by IVM was initially shown to be a successful treatment for infertile women with anovulatory PCOS,^43^, ^45^ and then for infertile women with high AFC.^111^, ^112^ In women with regular menstrual cycling ovaries, approximately 20 antral follicles were selected and carried through to the preovulatory stages of development during each menstrual cycle.^113^ Two or three waves of ovarian follicular development in this group of women during menstrual cycle have been reported based on daily transvaginal ultrasonography, challenging the traditional theory of a single cohort of antral follicles that grows only during the follicular phase of menstrual cycle.^114^, ^115^ Thus, in women with regular menstrual cycling ovaries, the significant advantage of priming with hCG alone before oocyte retrieval is to collect the mature oocytes from the leading or dominant follicles other than immature oocyte retrieval. It is important to know that the quality and early embryonic development competence of immature oocytes following IVM technology are not detrimentally impacted by the presence of the dominant follicle in the ovaries.^31^, ^32^, ^33^ Based on those findings, the clinical application of IVM technology has continued to evolve.

The consideration should be given in this group of women to natural cycle IVF and administration of hCG when the leading or dominant follicles reach a certain size. In such cases, one or two mature oocytes together with several immature oocytes can be retrieved at the time of oocyte retrieval, 36 h after hCG priming, as mentioned above, namely natural cycle IVF combined with IVM protocol was developed.^112^, ^116^ Therefore, it seems that the attractive possibility for increasing the success rate of natural cycle IVF treatment is the combination of in vivo matured oocytes and immature oocyte retrieval. If the mature oocytes from the dominant or leading follicle and the immature oocytes from the small follicles are all collected, the chances of a pregnancy may be greatly increased when the immature oocytes are to mature in vitro and can produce more viable embryos.

It may be important to prevention premature ovulation from the dominant follicle in the natural cycle IVF combined with IVM treatment. In our experience, we have administered 10 000 IU hCG 36 h before oocyte retrieval, when the size of the dominant follicle reached 10–14 mm in diameter. Clinical pregnancy rate may reach about 45%–50% per embryo transfer, following natural cycle IVF combined with IVM treatment in a selected group of women.^112^ Also, more than half of all infertile women who seek out IVF treatment can be treated with natural cycle IVF combined with IVM or the treatment of IVM alone. When the natural cycle IVF combined with IVM treatment has been initially chosen, this can indicate that the treatment is an efficient option, especially for women under the age of 35 years.^111^ This can also indicate that natural cycle IVF combined with IVM treatment may be more desirable for patients that have poor response or failures of treatments of stimulated cycles.^117^ Interestingly, a recent report indicated that even with the presence or ovulation of the dominant follicle from the ovaries, this does not significantly influence the developmental and implantation capacity of immature oocytes retrieved from small follicles. Thus, further confirming that natural cycle IVF combined with IVM treatment is a promising treatment option for women without ovarian stimulation.^33^

Today, given the efficiency of IVF and improvements in the culture system, natural cycle IVF or mild stimulation may be more suitable for women undergoing IVF treatment. In contrast to conventional IVF treatment, the aim of mild stimulation is to develop safer and more patient‐friendly protocols where the risks of the treatment are minimized,^118^ suggesting that mild ovarian stimulation for IVF should be considered in all clinical scenarios as it is as effective as conventional IVF in terms of pregnancy outcome but also safer, better tolerated and less expensive.^119^ The mild stimulation or modified natural cycle IVF is defined as administration of low‐dose exogenous gonadotropins, a shorter duration in gonadotropin‐releasing hormone antagonist cotreated cycles, or when oral compounds, such as Clomiphene Citrate (CC), are used for ovarian stimulation, with the aim of retrieving more oocytes.^120^, ^121^ Interestingly, the mild stimulation using CC in combination with low doses of gonadotropins can also be considered as a realistic option for patients undergoing IVF with a good prognosis.^122^

Although treatments with natural cycle IVF combined with IVM together with IVM‐alone treatments can achieve acceptable pregnancy and implantation rates in more than 50% of infertile women, there can be technical difficulties in immature oocyte retrieval from the small follicles after the retrieval of mature oocytes from dominant and leading follicles.^111^ Therefore, we have proposed the modified treatment based on natural cycle IVF combined with IVM, namely mild stimulation IVF combined with IVM treatment.^16^, ^17^ The methodology describes that the leading follicles will be aspirated first, and then the sub‐leading follicles will be aspirated. The mature oocytes can be inseminated with IVF, if the parameters of sperm quality are normal, otherwise ICSI can be applied.

To identify the immature oocytes, both the size of follicles derived from and the morphology of expansion of cumulus–oocyte complexes (COCs) must be analyzed. The COCs with few inner layers of compacting cumulus cells with a good pattern of outer layer cumulus expansion appeared as they can be denuded 4–6 h after culture in fertilization medium or IVM medium to determine oocyte maturity. When the oocytes have matured, they can be inseminated by IVF or ICSI, and the remaining immature oocytes can be cultured overnight in IVM medium. Those immature oocytes will be checked again 24 h after IVM for possible insemination. The mature oocytes can be inseminated by ICSI using the sperm sample prepared on the previous day, which needs to be maintained at room temperature with a tightly capped tube.^123^

**FIGURE 2:**
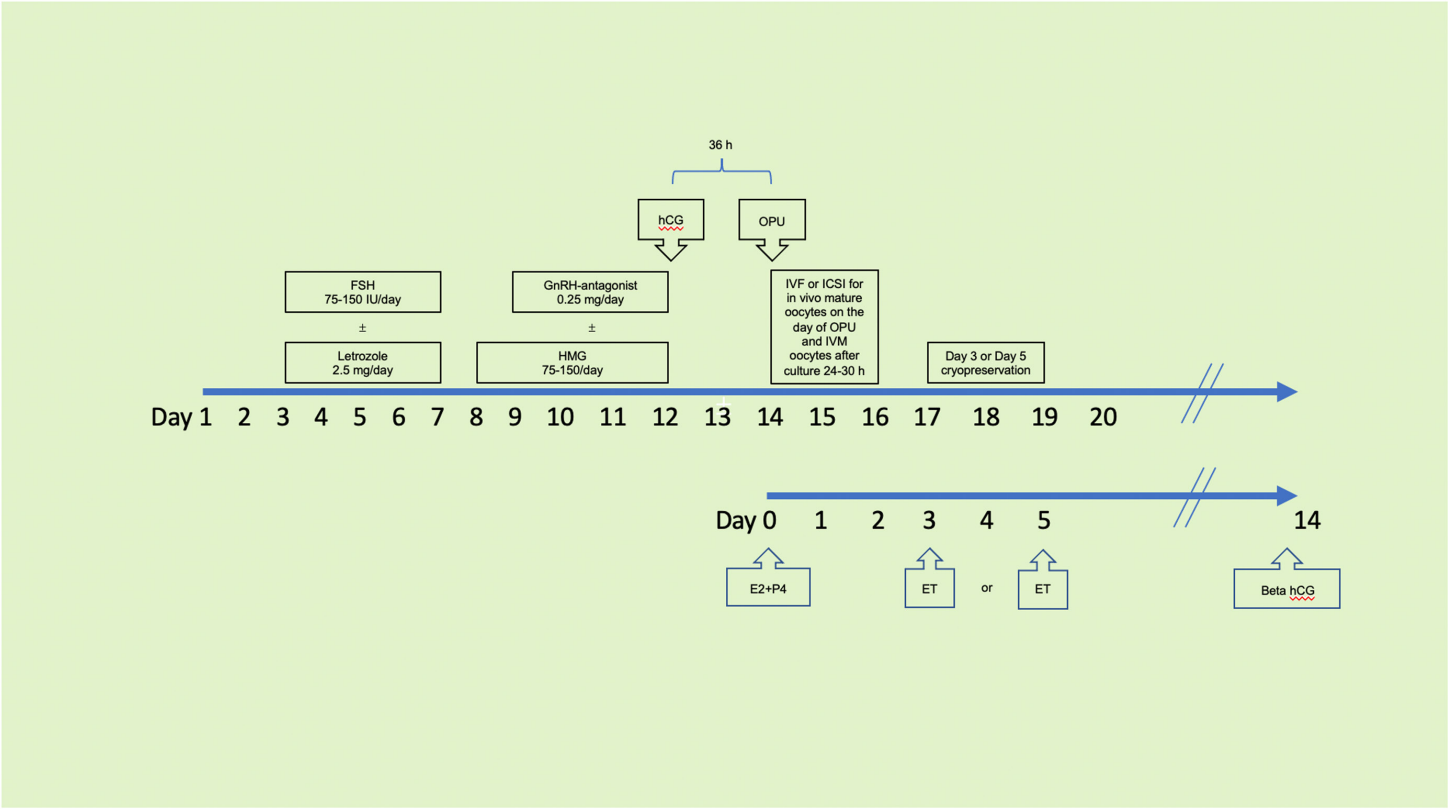
The protocol of the modified natural cycle IVF or mild stimulation cycle IVF combined with IVM of immature oocyte treatment. E2, estradiol; ET, embryo transfer; ICSI, intracytoplasmic sperm injection; IVF, in vitro fertilization; IVM, in vitro maturation; OPU, oocyte pick‐up; P4, progesterone.

A protocol for the modified natural cycle IVF or the mild stimulation cycle IVF combined with IVM treatment has been developed (Figure 2). Our preliminary data revealed the effectiveness of the modified natural cycle IVF or mild stimulation IVF combined with IVM treatment approach.

## 6 CONCLUSION

IVM technology is a potentially useful technique for infertile women and fertility preservation. However, the proper storage of maternal mRNA during oocyte growth is the key point for oocyte maturation through meiosis to generate mature and haploid oocytes because the oocyte can only use the stored mRNA to synthesize new proteins to support subsequent early embryonic development. Clinical IVM refers to maturation in culture of fully grown immature oocytes at different stages that may or may not have been exposed to gonadotropins.

The source of immature oocytes can be considered the critical factor for IVM. Apart from the size and phase of follicles, the quality of immature oocytes is directly related to the age of the woman. Immature oocytes may be retrieved from women after priming with or without the use of FSH, hCG or a combination of both FSH and hCG. Successful pregnancy rates with IVM technique seem to be correlated with the number of immature oocytes obtained. With the source and culture course of immature oocytes, various IVM protocols have been proposed. IVM of immature is profoundly affected by culture conditions, but no breakthrough has yet been made by improving the IVM medium itself.

The clinical application of IVM technology has continued to evolve, namely natural cycle IVF or mild stimulation IVF combined with IVM of immature oocytes. This treatment represents a viable alternative to the conventional stimulation IVF cycle treatment as it may prove to be an optimal first‐line treatment approach.

## CONFLICT OF INTEREST STATEMENT

The authors declare no conflict of interest.
